# Life Course Cognability: A Mixed Methods Study of Neighborhoods and Cognitive Health

**DOI:** 10.1093/geroni/igaf122.542

**Published:** 2025-12-31

**Authors:** Michael Esposito, Jessica Finlay

**Affiliations:** University of Minnesota, Minneapolis, Minnesota, United States; University of Colorado Boulder, Boulder, Colorado, United States

## Abstract

This mixed methods study extends the concept of Cognability -- a new theoretical framework for understanding how neighborhood environments shape cognitive health -- to investigate specific built and social environmental features that impact cognitive health across the adult life course. Current understanding of which neighborhood factors matter when across adulthood is limited. We will conduct a sequential mixed methods approach (QUAL -> QUANT) to identify neighborhood risk and protective factors influencing cognitive health. First, qualitative interviews with 240 adults across four metropolitan areas (San Francisco, Denver, New York, Atlanta) will investigate how individuals of different ages navigate local environments to engage in cognitive health-promoting behaviors. Second, we will utilize these insights to construct sophisticated quantitative models in a coordinated integrated data analysis of four national longitudinal cohort studies (AddHealth, ACL, REGARDS, HRS) spanning adolescence to late age, with geographic data for nearly 90,000 unique respondents. Using structural nested mean models to unravel tricky counterfactual histories, we will assess whether amenities, services, and hazards identified through our qualitative data systematically predict cognitive function trajectories nationwide. This project will advance understanding of Alzheimer’s Disease and Related Dementias (AD/ADRD) by identifying modifiable environmental factors across adulthood, not just in later life. By integrating qualitative insights with sophisticated quantitative methods, we aim to develop actionable evidence to inform community interventions that support cognitive health behaviors, address geographic barriers to health outcomes, and advance much-needed ‘exposome’ research.

